# Understanding social situations: study protocol for a randomized controlled trial evaluating a novel social cognitive training versus modified problem-solving training for people with psychosis

**DOI:** 10.3389/fpsyt.2024.1440476

**Published:** 2024-08-22

**Authors:** Joanna M. Fiszdon, Morris D. Bell, Daniel Fulford, David L. Roberts, James Dziura, Lori Parente, Alexis Nasse, Jimmy Choi

**Affiliations:** ^1^ Psychology Department, VA Connecticut Healthcare System, West Haven, CT, United States; ^2^ Department of Psychiatry, Yale University School of Medicine, New Haven, CT, United States; ^3^ Department of Occupational Therapy and Department of Psychological & Brain Sciences, Boston University, Boston, MA, United States; ^4^ Deparment of Psychiatry, UT Health Sciences Center, University of Texas, San Antonio, TX, United States; ^5^ Yale Center for Analytical Sciences and Division of Biostatistics, Yale University School of Public Health, New Haven, CT, United States; ^6^ Olin Neuropsychiatry Research Center, Institute of Living at Harford Healthcare, Hartford, CT, United States

**Keywords:** schizophrenia, treatment, cognition, ecological momentary assessment, social functioning, veteran

## Abstract

**Background:**

Psychotic spectrum disorders (PSD) are associated with poor social function. In this study, we investigate which of two different types of 2-month long training courses is more effective in improving day-to-day interactions and quality of life.

**Methods/design:**

Participants with psychotic spectrum disorders will be randomly assigned to one of two training courses. Social functioning, everyday activities, social cognition and symptoms will be assessed at multiple timepoints, including baseline, treatment midpoint, end of treatment and 2-month follow-up. One training focuses on how to make good judgments about what other people may be thinking or feeling in social situations, and why people might act in certain ways in different situations. The other training focuses on different strategies for handling everyday problems and stressors. Both trainings are done in one-on-one sessions with a research staff member. There will be 16-20 training sessions, each about 45-60 minutes long. The investigators will ask participants to attend 2 training sessions per week, so the total training time should be about 2 months.

**Clinical Trials Registration:**

PROSPERO, identifier NCT04557124

## 1 Introduction

Functional disability is a core, defining feature of psychotic spectrum disorders (PSD) (1), with impairments in social function impending recovery and leading to significant social isolation (2). These impairments are present prior to illness onset (3), persist throughout different phases of the illness (4) and are more severe in PSD than in other serious mental illnesses (5). Poor social function in PSD has also been linked to significant impairments in both cognition (6–8) and social cognition (9–11), with a growing body of research suggesting that social cognition contributes unique predictive power and may mediate the relationship between cognition and social function (9, 12–19). For these reasons, social cognition has been proposed as a proximal treatment target for interventions aimed at improving social function and promoting recovery in people with PSD (7, 20, 21).

A number of social cognitive treatments for PSD have been developed. On the whole, published trials evaluating these interventions have shown some promise, with large, consistent effects on lower order skills like emotion recognition, more modest, somewhat less consistent effects on some higher order skills, and limited evidence of generalization to social functioning (21–25). A closer look at the existing evidence base suggests caution in drawing any conclusions and a need for more research. For example, in a recent systematic review of randomized controlled trials (24), only 3 of 32 studies reviewed met what was considered a minimum threshold for study quality, with the remaining studies at moderate to high risk of bias due to a combination of variables such as lack of randomization, unblinded assessors, lack of fidelity assessments, and comparison to usual treatment. Moreover, study samples sizes were for the most part quite modest (~20/arm) and approximately 90% of the trials were underpowered to consistently obtain reported results. Additional common critiques of the current state of social cognitive treatment research include lack of follow-up to determine durability of treatment effects, near absence of psychometrically sound social cognitive measures and heterogeneity of measures across different trials, no independent replication, infrequent assessment of generalization to everyday functioning, and little focus on factors that may influence response to training (21–23, 25–27). In sum, there is a clear need for more rigorous trials that use psychometrically sound measures, include social function outcomes, assess durability, and evaluate who is most likely to respond to the intervention and under what conditions.

In the current manuscript, we describe the protocol for a large rigorous randomized controlled trial to test the efficacy of a novel social cognitive training called Understanding Social Situations (USS). The training leverages successful methods from bottom-up cognitive remediation to reduce cognitive load and aid in acquisition of social cognitive skills. We proposed to randomize 120 Veterans with PSD to 2 months of USS or an active control (AC) intervention matched for duration, therapist contact, and mode of delivery. Key social functioning outcomes will be measured using a multi-method approach of self-report, role-play, and experience sampling, conducted pre-intervention, post-intervention, and at 2 month follow-up, with an additional limited assessment at treatment mid-point. Our aims are as follows:

Primary Aim:

Aim 1: Examine the efficacy of USS in improving social functioning.

Secondary Aims:

Aim 2: Examine the efficacy of USS in changing real-world social behaviors.

Aim 3: Examine efficacy of USS in improving social interaction skills.

Aim 4: Examine durability of USS effects on clinician and self-rated social functioning, real-world social behaviors, and social skills.

Exploratory Aims: Examine mechanisms of USS effects.

A. Target engagement and validation: examine impact of USS on a measure of USS content learning (USS Skills Test) and relationship between content learning and improvement in social functioning.

B. Personalization: explore baseline cognitive, treatment dose, symptom clusters and demographic variables as potential moderators of USS efficacy.

## 2 Materials and methods

### 2.1 Study design

**Figure 1:**
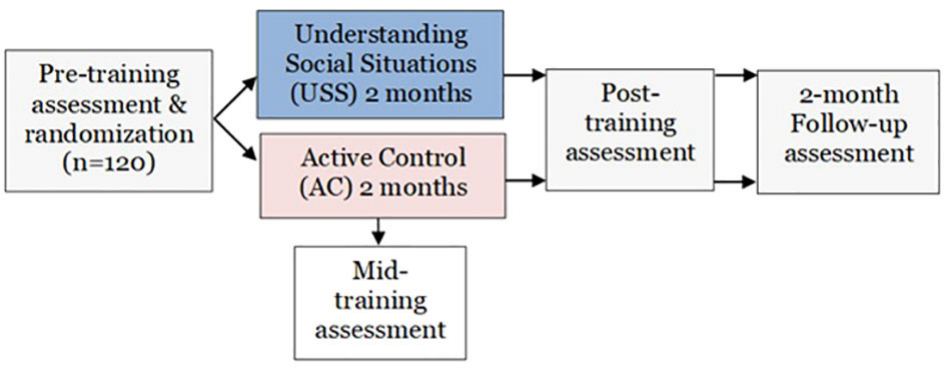
Study schematic.

We propose a single-site, randomized, controlled trial investigating the efficacy of Understanding Social Situations (USS) in improving social functioning in participants with psychotic spectrum disorders. One hundred twenty participants will be randomized to two months of individually administered USS or a matched active control training. Comprehensive assessments will occur at baseline, end of 2-month training phase, and two-month follow-up, with an additional assessment of the primary outcome and of USS content-related skill (treatment target) at training mid-point. See **Figure 1** for study schematic.

We will examine the efficacy of USS on social functioning (primary outcome), real-world social interactions, and social skills. We will also explore moderators and mediators of treatment effects, specifically the impact of baseline variables on treatment efficacy and the relationship between USS content knowledge test and social functioning change. The study is sponsored by VA Office of Research and Development and the protocol has been approved by the VA Connecticut Healthcare System Institutional Review Board. All participants will provide informed consent. This clinical trial has been registered: NCT04557124.

### 2.2 Participants

Participants will be Veterans with a psychotic spectrum diagnosis (PSD). Specific inclusion/exclusion criteria are as follows: diagnosis of psychotic disorder (e.g. schizophrenia, schizoaffective disorder, delusional disorder, psychosis NOS, affective disorder with psychotic features); no prior exposure to USS training, age 18 and over; not meeting criteria for substance use disorder in past 30 days; psychiatric stability as evidenced by minimum of 30 days since last psychiatric hospitalization and since last change in psychiatric medications; no evidence of developmental disability in chart or on baseline assessment; no severe, uncorrected auditory/visual impairment; no diagnosis of medical or neurological illness known to impair brain function including dementia, presence of seizures, history of head trauma with loss of consciousness > 1hr, or clear cognitive sequelae from other illness or injury, per medical chart review; fluency in English; ability to provide legal written informed consent (i.e. the participant does not have a conservator); not currently enrolled in another treatment study targeting, or expected to impact, functioning.

### 2.3 Study procedures

Veterans who express interest in the study will initially be pre-screened by phone. Those who meet preliminary screening criteria and are interested in study participation will then undergo informed consent procedures, followed by a more thorough screening and baseline assessment. Those who meet all eligibility criteria and consent to study participation will be randomized to receive either USS or AC in a 1:1 ratio using a permuted block design with variable block size, stratified by baseline social functioning (Social Functioning Scale score > 100). The randomization scheme will be computer-generated by the study statistician.

Following written informed consent and baseline assessment, participants will be provided with smartphones and oriented to the EMA procedures. This will include practice with receiving Qualtrics links to EMA surveys, navigating the questionnaires on their smartphones, and a comprehension check of survey questions and responses. For those who do not currently own smartphones, orientation will also include basic use and charging of the smartphone. Once they have completed baseline EMA surveys, they will be randomized into one of two training conditions: Understanding Social Situations (USS) or a matched Active Control (AC). Training sessions will be delivered approximately twice per week, over approximately 8-10 sessions. For both USS and AC, participants will be asked to attend two cycles of the training, for a total of approximately 16-20 sessions over about two months. This approach mirrors many real-world clinical settings where Veterans undergo multiple cycles of an intervention, is intended to further compensate for cognitive impairments, and is intended to assure that training content is learned and consolidated. Participants will have the option of doing training sessions face-to-face, or via video conference, and all attempts will be made to reschedule missed sessions. In order to control for treatment intensity, we will allow for no more than 3 months to complete the training, at which timepoint we will discontinue training and initiate post-testing assessments, whether or not the participant has completed two cycles of the training. Training sessions will be recorded for later fidelity ratings. While participants will not be blind to treatment condition, they will be blind to study hypotheses and we will promote equipoise by presenting both interventions as targeting everyday function. Comprehensive assessments will be conducted at baseline and repeated at two months (end of active phase), and four months (two-month follow-up from end of active phase). An additional assessment for the primary outcome (Social Functioning Scale) and hypothesized moderator (USS Skills Test) will occur at one month (midpoint through the active phase). Participants will be paid as follows: $50/each for baseline, post-training and 2-month follow-up assessments; $10 for midpoint assessment; $1/each EMA survey assessment ($4/day); $10/training session. They will also be able to keep the study-provided smartphones and those completing at least 80% of EMA surveys will be entered into a raffle for an additional $100 payment.

### 2.4 Interventions

A research assistant will be trained on the delivery of both interventions. Training will consist of reading the USS and the AC training manuals, reviewing USS training videos, and hands-on demonstration of both trainings by study PI, followed by practice in intervention delivery with feedback from PI. Training progress will also be reviewed in weekly lab meetings.

#### 2.4.1 Understanding social situations experimental intervention

USS was developed by the PI and collaborator Roberts under a NIMH R34 grant (28). It was developed to train higher-order social cognitive skills. Training content was largely adapted from successful lab-based experimental interventions targeting theory of mind and attributional bias. Given that significant cognitive impairments in people with psychosis can limit skill acquisition, USS was uniquely developed to lessen cognitive load by relying on delivery techniques that have previously been successfully used in cognitive remediation including scaffolding, hierarchical training, massed drill and practice, performance-based increases in task difficulty, and verbal mediation. Additional techniques include motivational enhancement and use of homework to promote bridging to real-world situations. Complex skills are trained by first practicing their individual components. There are four training modules (see **Table 1**) that are administered over 8-10, individual, 45-60 minute sessions. The skills taught in USS are designed to build on one another over the course of the training. Trainees first practice distinguishing social facts from guesses, with trainers reinforcing the (somewhat simplistic) idea that anything that cannot be directly perceived (heard, felt, seen), is not a fact, and therefore is subject to different interpretations (i.e., it is a guess). Trainees then practice distinguishing good guesses from bad guesses using the criteria that good guesses are accompanied by many supporting facts and few, if any facts that could support different guesses. Next, trainees practice quantifying their confidence in the quality of a guess (its likelihood of being true) as a function of the amount and type of facts that support it. This is followed by practice in gathering information to make good (i.e., fact-based) inferences about other people’s thoughts and intentions. Trainees learn to use verbal elaboration to more deeply process social stimuli in order to improve the quality of their judgments. The final module of USS does not build on the previous content, but rather uses a laboratory fill-in-the-blank task to implicitly induce positive social interpretive bias in trainees. The intervention techniques used in USS are adaptations of existing treatments or laboratory manipulations (31, 32, 36–38). Training stimuli consist of photos, videos, cartoons, written vignettes, and audio clips of mostly social situations. Each session consists of summary of prior session, review of homework, new content, and homework.

**Table 1:** Understanding Social Situations (USS) training components.

**UNDERSTANDING SOCIAL SITUATIONS (USS) TRAINING** Techniques employed throughout USS content include hierarchical training, massed drill and practice, breaking skills into subcomponents, graded increases in task difficulty, scaffolding, errorless learning, verbal mediation, modeling, minimizing memory load via visual cues, and use of homework to promote bridging to real-world situations. The first three USS modules contain hierarchical difficulty levels, with task difficulty manipulated by adjusting response format, plausibility of foils, stimulus ambiguity, valence, self-relevance, etc. Progress through the training is tailored to individual performance in order to provide an optimal level of challenge while minimizing frustration. Sessions are highly structured and include homework, review of prior session’s content, modeling and practice of new skills, and assignment of new homework.
**Psychoeducation and motivation enhancement:** The trainee is provided with a rationale for engaging in the ensuing training (to better understand social situations which will help in getting along well with others). Brief video vignettes of social problems are reviewed and the trainee’s own experiences with situations where s/he had difficulty figuring out what the other person was thinking or feeling are discussed. The trainee identifies a specific goal pertaining to his or her social life. An overview of the skills to be trained is provided.
**Module 1 Separating Facts from Guesses**: Training focuses on distinguishing between observable behavior and inferences about thoughts, feelings and social meanings. Training progresses from identifying what are tangible facts, and distinguishing them from guesses, particularly about others’ mental states. Training further progresses to distinguishing between good, fact-based guesses versus bad guesses that have little or no support. (Techniques adapted from Social Cognition Interaction Training, SCIT, 29, 30).
**Module 2 Probability Judgments and Not Jumping to Conclusions**: Training focuses on developing skills to evaluate the quality of guesses based on how much information is available to support guesses. Training progresses from rating guesses as good or bad, to rating the relative likelihood of multiple guesses about a single situation, to re-rating quality of different guesses as more information is provided about the situation (techniques adapted from Moritz & Woodward (31).
**Module 3 Determining Others’ Mental States and Intentions**: Training focuses on using verbal mediation to process temporal sequences of social events and identify information in support of various guesses about a character’s current or future intentions. Training progresses from evaluating individual stimuli to integrating information from multiple stimuli to making guesses about characters’ mental and emotional states and intentions (techniques adapted from Sarfati and colleagues (32, 33).
**Module 4. Inducing Positive Interpretive Bias:** Cognitive bias modification training. Goal of this module is for trainees to develop an automatic bias toward interpreting ambiguous social events in a positive manner. Trainees practice by completing very brief written stories about themselves in social situations, with each story resolving in a favorable way. Unlike other modules, there are no difficulty levels (techniques adapted from Constans and colleagues (34, 35).

#### 2.4.2 Moving forward intervention, active control

The active control intervention was selected to match USS on interaction with study staff, treatment duration and intensity, and delivery format. “Moving Forward: Overcoming Life’s Challenges” is a free, web-delivered training developed by the VA as part of the Integrated Mental Health Strategy initiative to expand access, ensure quality of care, promote resilience and build better behavioral health systems (https://www.veterantraining.va.gov/movingforward). The training is based on Problem Solving Therapy (39), an evidence-based cognitive-behavioral approach to developing problem solving skills to effectively cope with stressors (40). It consists of 8 modules focused on what the training is, how it may be helpful, how to solve problems under stress, steps of problem solving, and how to apply what was learned to daily life. Modules 1, 2 and 3 offer an introduction to the program, what to expect and what the training works toward. Modules 4 and 5 target managing and overcoming stress. Modules 6, 7, and 8 focus on the step-by-step process for solving problems and how to apply this to daily life. In preparation for the current study, we thoroughly reviewed and timed module content, added homework assignments, and developed manualized procedures for how this normally self-administered program can be delivered by a study trainer in 45-60 minute sessions.

### 2.5 Assessments

Baseline assessments will consist of demographic, intelligence, psychiatric, cognitive, social function and knowledge of USS training content measures. Post-training and 2-month follow-up assessments will mirror baseline assessments, with the exception of diagnostic interviews and IQ estimate, which will only be administered at baseline. An additional assessment of the primary outcome, Social Functioning Scale, and of the proposed mediator of treatment effects, USS Skills Test, will occur at treatment mid-point. Please refer to **Table 2** for details.

**Table 2:** Assessment timeline.

Assessments Timeline	Duration	Measure type	Pre	Mid	Post	FU
Demographics/Psychosocial	10 min	sample characteristics, potential moderators	X			
Wechsler Abbreviated Scale of Intelligence (WASI, 2 subtest)	20 min	IQ estimate, sample characteristics, potential moderator	X			
Structured Clinical Interview for DSM (SCID)	60 min	diagnostic, sample characteristics	X			
Positive and Negative Syndrome Scale (PANSS)	30 min	symptom severity, sample characteristics, potential moderator	X		*X*	*X*
Matrics Consensus Cognitive Battery (MCCB)	60 min	cognition, sample characteristics, potential moderator	X		X	X
Social Functioning Scale (SFS)	15 min	social function primary outcome, potential moderator	X	X	X	X
7-day EMA assessment	3min/each assessment (total 12 min/day)	social function secondary outcome	X		X	X
Social Skills Performance Assessment (SSPA)	10 min	social function secondary outcome	X		X	X
Acceptability feedback	5 min	Feedback on acceptability and suggestions for improvement		X	X	
USS Skills Test	5 min	social cognition training target, potential moderator	X	X	X	X

#### 2.5.1 Intelligence

The Wechsler Abbreviated Scale of Intelligence (WASI, 41), 2-subtest estimate) will be used to obtain estimates of current intelligence (IQ Estimate score). The WASI 2-subtest IQ estimate correlates highly (r=.87) with the Wechsler Adult Intelligence Test (42), the most commonly used and accepted measure of intellectual function. It also has high internal consistency reliability (r=.93 for adult sample), and 2-12 week test-retest stability (r=.85). The WASI will be used to characterize the study sample and screen for intellectual disability (IQ < 70) at baseline.

#### 2.5.2 Symptoms

The Structured Clinical Interview for DSM-V (SCID, 43) will be used to confirm psychotic spectrum diagnoses. The SCID is the most commonly used semi-structured interview for obtaining DSM-V diagnoses. Modules A through E will be used to determine presence of psychotic and mood syndromes, substance use history, and differential diagnosis. The Positive and Negative Syndrome Scale (PANSS, 44) will be used to characterize participants by assessing the presence and severity of psychiatric symptoms. The PANSS is an interviewer-rated scale indexing the core symptoms of psychosis as well as a broad range of general psychiatric symptoms including depression and anxiety. Each symptom is rated on a Likert-type scale ranging from 1-7, for total score range of 30 to 120. Initial reports using this scale provide evidence of good internal reliability for the three subscales (alpha =.73 to.83), with adequate test-retest reliability over 3-6 month inpatient phase (r=.60 to.80) and good interrater reliability (r=.83 to.87). Within our research group, ICC’s against gold standard, study PI, range from.85-.97 for PANSS components. For initial exploratory analyses of symptom severity as a potential moderator of treatment effects, PANSS Total Score will be used. As warranted, subsequent analyses may examine the Positive, Negative, and General symptom subscales.

#### 2.5.3 Cognition

Cognition will be assessed using the MATRICS Consensus Cognitive Battery (MCCB, 45). The MCCB was developed by an expert panel of researchers, under NIMH contract, as a broad yet sensitive measure to assess cognitive change in treatment studies. The MCCB includes assessment of 7 domains: speed of processing, attention/vigilance, working memory, verbal learning, visual learning, reasoning and problem solving, and social cognition. Four-week test-retest reliability for individual cognitive domains ranges from ICC =.71-.85, and many of the tests include alternate forms to reduce ceiling effects. While an overall composite score is available, in recognition of differences between neurocognitive and social cognitive impairments, it is becoming more and more common in studies of neurocognitive function in psychosis to calculate a 6-domain composite score, omitting the social cognitive domain (46). This composite score will be used for initial exploratory analyses of cognition as a potential moderator of treatment effects.

#### 2.5.4 Social functioning primary outcome

Social functioning will be measured using the interviewer-administered Social Functioning Scale (SFS, 47), which assesses social functioning across seven domains: social engagement/withdrawal, interpersonal behavior/communication, prosocial activities, recreation, independence—competence, independence—performance, and employment/occupation. The SFS is one of the best known measures of social functioning in schizophrenia, and was one of two social function measures nominated by experts and selected by a RAND panel for a large-scale investigation of measures to assess real-world outcomes (48) based on its psychometric properties, sensitivity to change, relationship to symptoms, and comprehensiveness. Importantly, our pilot data indicates that the SFS is sensitive to the effects of the USS intervention. Total score will be used as our primary outcome. The SFS was originally validated with 334 outpatients with schizophrenia and their relatives, along with 100 healthy controls and their relatives. Coefficient alpha was.80, with inter-rater reliability of.94 and rater self-report correlation of.78. Item-total correlations were generally in the.70 range, suggesting high internal consistency. Construct validity was supported via factor analysis revealing a single factor (eigenvalue 3.96), accounting for 57% of the variance. Criterion validity was supported by the scale’s ability to distinguish between clinical, sibling and healthy control samples, and between employed and unemployed subgroups.

#### 2.5.5 Social functioning secondary outcome

Social Skills Performance Assessment (SSPA, 49) is a role-play measure of social skill ability. It consists of two, 3-minute, structured role plays (tenant meeting a new neighbor; tenant calling landlord to request repair). The role plays are audio-taped and rated on a number of characteristics including interest/disinterest, clarity, social appropriateness, negotiation ability, and overall conversation, among others. Interrater agreement for the scale is high (ICC=.91), as is test-retest reliability (r=.92, 1 week). The SSPA has good convergent and divergent validity, and is sensitive to impairments accompanying psychosis. For our analyses, scores from the two role-plays will be summed into a single total score. Smartphone-delivered Ecological Momentary Assessment (EMA), an experience sampling method, will be used to capture information about the extent and type of social interactions, along with participants’ dispositions toward and subjective appraisals of these interactions. Questions were mostly adapted from earlier EMA work with similar populations (50–52), and include prompts about the frequency and nature of social interactions, enjoyment level, perceived clarity of communication and confidence in understanding the other person’s intent, and anticipation of future social interactions. EMA methodology has the advantage of reducing memory demands and/or recall bias and providing an ecologically valid measure of day-to-day experiences. EMA questions will be administered via smartphone 4 times per day for a 7-day period at baseline, immediately after the end of the 2-month active phase, and at a follow-up two months following end of the active phase.

#### 2.5.6 Training target

Consistent with the experimental therapeutics approach, we will not only evaluate outcomes of interest, but also the potential impact of the intervention on the hypothesized treatment mechanism. In this case, we hypothesize the mechanism to be social cognitive skill, indexed by learning of content taught during USS. Hence, we will use the USS Skills Test to index target engagement. The USS Skills Test is a 22-item measure assessing knowledge of principles and skills taught during the training (28). Items on the USS Skills Test are similar to (though not identical) to those used in the USS training stimuli, and as such, should provide a proximal measure of training effects. Our pilot data indicates that the USS Skills Test is sensitive to training effects.

#### 2.5.7 Acceptability

At the end of each round of training, we will also administer a brief acceptability questionnaire, querying whether the participants found the training helpful/useful, whether they thought it helped them better understand social situations and other people, what they liked most/least about the training, and what suggestions they had for how to improve the training.

### 2.6 Statistical analyses

No imputation of missing data will be performed in the primary and secondary analyses. Diagnostic tests and sensitivity analyses will be performed. Parametric distributional assumptions will be checked. If assumptions fail, other distributions will be considered prior to transformations and non-parametric methods. For all analyses, two-sided significance tests will be implemented and will be performed using SAS v9.4 (53).

#### 2.6.1 Analysis of primary outcome, SFS (aim 1)

The primary objective of the analysis is to demonstrate that USS will improve social functioning at the end of training more than active control in participants with PSD. The primary outcome (total scores on the SFS) will be assessed prior to initiation of intervention (pre), the training mid-point (mid), the end of training (post) and 2 months following the end of training (FU). Likelihood-based ignorable analysis using a linear mixed model will be used to compare social functioning between groups (54, 55). The primary advantage of the repeated measures linear mixed model when compared to commonly used methods such as complete case analysis and single imputation (e.g. last observation carried forward) is its flexibility in handling missing data. This analysis will assume that missing data occurs at random (i.e. MAR, not informative). The inclusion of pre, mid and FU outcome data in the model will assist in meeting this assumption. Furthermore, we will evaluate patterns of missing data as well as determine baseline characteristics that are predictive of dropout. If identified, these characteristics will be included in the model to meet the MAR assumption. The mixed model will include fixed effects for intervention (USS vs. active control), time (mid, post, FU), and the interaction of intervention with time. An additional fixed effect will be included for baseline SFS at pre. An unstructured covariance pattern will be used to accommodate correlation from repeated measures. A linear contrast will be used to estimate intervention group differences and 95% confidence intervals at the post assessment.

#### 2.6.2 Analysis of real world social behaviors (aim 2)

Ecological momentary assessment (EMA) analyses will be conducted using a multilevel modeling approach (56). Analyses handle data estimation with a restricted maximum likelihood approach. Data will be organized hierarchically, with within-person/random EMA prompts across the study period nested within people. Random (within-person) coefficients will be estimated for each person at Level 1, while fixed (between-person) coefficients will be estimated at Level 2. Within-person variables will be centered at Level-1 and between-person variables will be grand mean centered at Level-2 (57–59). We will examine associations between [e.g., attributions] and [e.g., number of interactions] at Level 1, and fit models with fixed [e.g. group, age, gender] main effects at Level 2. We will also examine [L1] and [L2] cross-level interactions.

#### 2.6.3 Analysis of social interaction skills (aim 3) and durability (aim 4)

The secondary outcome social interaction skills, assessed by SSPA, will be compared between USS and control using a repeated measures linear mixed model similar to that described in the analysis of the primary outcome. Durability will also be compared between USS and control using the mixed model. For primary and secondary outcomes, linear contrasts will be used to estimate intervention group differences and 95% confidence intervals at the 2-month FU assessment.

#### 2.6.4 Analysis of mediation (exploratory aim A)

We will explore content learning (USS Skills Test) as a potential mediator of the relation between intervention and changes in social functioning. Direct and indirect effects will be estimated using a structural equation model. Mediation (i.e. indirect effects) will be tested using the bootstrapping approach (60), with 10,000 bootstrapping samples used to estimate the confidence intervals of the indirect effects.

#### 2.6.5 Analysis of moderation (exploratory aim B)

Heterogeneity of treatment effects (HTE) for the primary outcomes will be explored in subgroups of participants based on baseline characteristics including cognition, symptom clusters, illness characteristics, medication dose (chlorpromazine equivalents) and demographic variables. These subgroup analyses are aimed at determining whether there is differential effectiveness of the interventions among participant subgroups. Evidence of HTE will be based on tests of interaction within the longitudinal model structure described above.

### 2.7 Sample size

The primary hypothesis is that USS will result in greater total SFS scores immediately following training (i.e. ‘post’ assessment) compared to control. We have powered our study to detect what we consider a clinically meaningful, moderate effect on this measure. Given the following: 1) power of 90%, 2) a two-sided 0.05 significance level, 3) a standard deviation for total SFS score of 13.1 (from our preliminary data), and 4) a 1:1 intervention allocation, a sample size of 46 subjects per group will be required to detect a 9 point difference (i.e. moderate effect size d=0.69) between USS and control in total SFS score at the post assessment. A total of approximately 120 participants will be enrolled to accommodate up to 20% dropout, which exceeds our actual anticipated dropout rate based on prior studies. Should drop-out be higher than anticipated, we will increase enrollment as necessary.

## 3 Anticipated results and discussion

In the current protocol, we seek to evaluate the efficacy of Understanding Social Situations (USS), a novel social cognitive training targeting higher-order social cognitive functions while leveraging successful bottom-up methods from cognitive remediation in order to reduce cognitive load and aid with skill acquisition. Using a rigorous RCT design, we plan to compare USS to an active control condition (AC) matched for modality, delivery format, duration, intensity, and therapist contact. We hypothesize that compared to AC, USS will be associated with greater improvements in social functioning, as assessed by both the Social Functioning Scale, real-world social behaviors captured using EMA, and social interactions skills as measured using the UCSD Social Skills Performance Assessment. We further hypothesize that these group differences will be maintained at a 2-month follow-up.

Our protocol is unique in that while the treatment focuses on social cognitive skills, our outcome measures focus on social function. If we fail to find group differences on social functioning, some may argue that it will not be possible to determine whether this was due to the training itself being ineffective, or to social cognition not being an adequate treatment target when change in social function is the outcome of interest. However, given the suboptimal psychometric properties of most social cognitive measures, including those recommended for clinical trials (61), we elected to focus on the more relevant, clinically meaningful outcome. While refinement of social cognitive measures continues, we will nevertheless be able to examine whether participants learned the skills taught during the training, as indexed by their performance on the USS Knowledge Test.

We chose an active control as a comparator to USS, in order to reduce the likelihood of subject-expectancy effects and enhance the rigor of the study. We are aware that this sets a high bar for observing differential treatment effects. Nevertheless, we felt that in order for future validation and implementation of USS to be warranted, the intervention should be at least in some ways superior to training that is already available. Such an approach is in line with a more rapid and efficient pipeline of treatment development-to-implementation trials. We should also note that no prior USS data was available to estimate effect sizes for Aims 2-4, and hence those analyses may be underpowered.

Impairments in social function are a core feature of PSD (1, 5). Existing treatments fall short of significantly improving social and community functioning, and social re-integration is among the top treatment needs both consumers and clinicians feel are not adequately addressed by existing interventions (62, 63). There is a clear need for more rigorous trials of social cognitive interventions that use psychometrically sound measures, include social function outcomes, assess durability, and evaluate who is most likely to respond to the intervention and under what conditions, The current trial will provide just such information about the efficacy of USS. If effective, USS has the potential to promote rehabilitation and recovery efforts by meaningfully impacting the social lives and wellbeing of people with psychosis.

## 4 Conclusions

USS training is unique in using methods from bottom-up cognitive remediation to compensate for cognitive impairments and enhance the likelihood of learning and consolidating social cognitive skills; If effective, It may represent an important addition to existing social cognitive interventions, and particularly so for those with significant (neuro) cognitive impairments who may struggle with other approaches that rely more heavily on difficult, complex cognitive processes such as reflection, brain-storming, or discussion.

## Data Availability

The original contributions presented in the study are included in the article/supplementary material. Further inquiries can be directed to the corresponding author.
